# Peste des Petits Ruminants Infection among Cattle and Wildlife in Northern Tanzania

**DOI:** 10.3201/eid1912.130973

**Published:** 2013-12

**Authors:** Tiziana Lembo, Christopher Oura, Satya Parida, Richard Hoare, Lorraine Frost, Robert Fyumagwa, Fredrick Kivaria, Chobi Chubwa, Richard Kock, Sarah Cleaveland, Carrie Batten

**Affiliations:** University of Glasgow, Glasgow, UK (T. Lembo, S. Cleaveland);; School of Veterinary Medicine, The University of the West Indies at St. Augustine, Trinidad and Tobago, West Indies (C. Oura);; The Pirbright Institute, Woking, UK (S. Parida, L. Frost, C. Batten);; Tanzania Wildlife Research Institute, Arusha, Tanzania ( R. Hoare, R. Fyumagwa);; Ministry of Livestock and Fisheries Development, Dar es Salaam, Tanzania (F. Kivaria, C. Chubwa);; The Royal Veterinary College, University of London, London, UK (R. Kock).

**Keywords:** peste des petits ruminants, rinderpest, morbillivirus, serosurveillance, disease eradication, cattle, wildlife, Tanzania, viruses

## Abstract

We investigated peste des petits ruminants (PPR) infection in cattle and wildlife in northern Tanzania. No wildlife from protected ecosystems were seropositive. However, cattle from villages where an outbreak had occurred among small ruminants showed high PPR seropositivity, indicating that spillover infection affects cattle. Thus, cattle could be of value for PPR serosurveillance.

Peste des petits ruminants virus (PPRV) is a highly contagious morbillivirus (genus *Morbillivirus*, family *Paramyxoviridae*) that is closely related to rinderpest virus. PPRV primarily affects sheep and goats in Africa, Middle East, and Asia but can infect a wide range of other domestic and nondomestic species (*1*–*4*). For example, cattle have been found to be seropositive for the virus (*1*,*2*), and PPRV was isolated from subclinically infected cattle 3 weeks after virus transmission from experimentally infected goats (R.K. Singh, pers. comm.). This finding points to the need for further investigation of the role of cattle in peste des petits ruminants (PPR) disease outbreaks. In addition, little is known about natural patterns of PPR in free-ranging African wildlife. Morbilliviruses can switch hosts, and new ecologic niches created by the eradication of rinderpest may provide opportunities for PPR emergence in new hosts (*4*,*5*).

First identified in West Africa in the 1940s, PPR is now widespread across much of sub-Saharan Africa. High rates of death from PPR can have dramatic economic consequences, especially in rural African communities whose livelihoods rely on small ruminant livestock production (*6*). The potentially devastating effect of new introductions has raised considerable concerns for local and regional economies (*6*). PPR was first confirmed in Tanzania in 2008 in the country’s northern regions. However, the virus, which was introduced into Tanzania by southward spread from neighboring countries, was probably in Tanzania long before official confirmation of the disease (*7**,**8*).

Capitalizing on the momentum resulting from the eradication of rinderpest, international agencies, including the Food and Agriculture Organization and the World Animal Health Organization, have focused attention on PPR; the disease has been identified as the next livestock disease candidate for eradication (*9*), and large-scale PPR interventions are being implemented in much of Africa. Epidemiologic surveillance is critical in global disease elimination and was considered key to the eradication of rinderpest (*10*). Although participatory surveillance was essential in assessing the levels and effect of cattle vaccination (*10*), wildlife serosurveillance was the primary tool for detecting the presence or absence of circulating rinderpest virus in the final stages of the eradication process (*11*). Grassroots-level surveillance is also likely to play a critical role in PPR control (*7*). However, critical questions remain about the role that species other than sheep and goats may play in PPRV persistence (*2*,*3*,*12*); the value of serosurveillance in control efforts; and, more specifically, whether cattle and wildlife species are useful indicators of virus circulation. To address these questions, we carried out serologic investigations for evidence of PPR infection in cattle and wildlife in northern Tanzania. The cattle lived in mixed cattle–small ruminant livestock systems in an area where a PPR outbreak had occurred in 2008, and the wildlife populations lived in protected-area ecosystems across a broader geographic area.

## The Study

Serum samples from cattle living in close proximity to sheep and goats were available as part of epidemiologic studies involving randomly selected pastoralist households in Ngorongoro District, an area of the Serengeti ecosystem in northern Tanzania where the 2008 PPR outbreak had been confirmed (Figure) (*7*). The sampling was conducted in early to mid-2011 and included serum samples from cattle >3 years of age (as determined on the basis of incisor tooth eruption) and from cattle <2 years of age; the older cattle were alive during the 2008 outbreak, and the younger cattle were born after the outbreak (Table 1). In the area, large-scale PPR vaccination of sheep and goats was initiated in early 2011 but did not include cattle. The last rinderpest vaccination campaign in Tanzania was carried out in 1997 (*13*).

**Figure:**
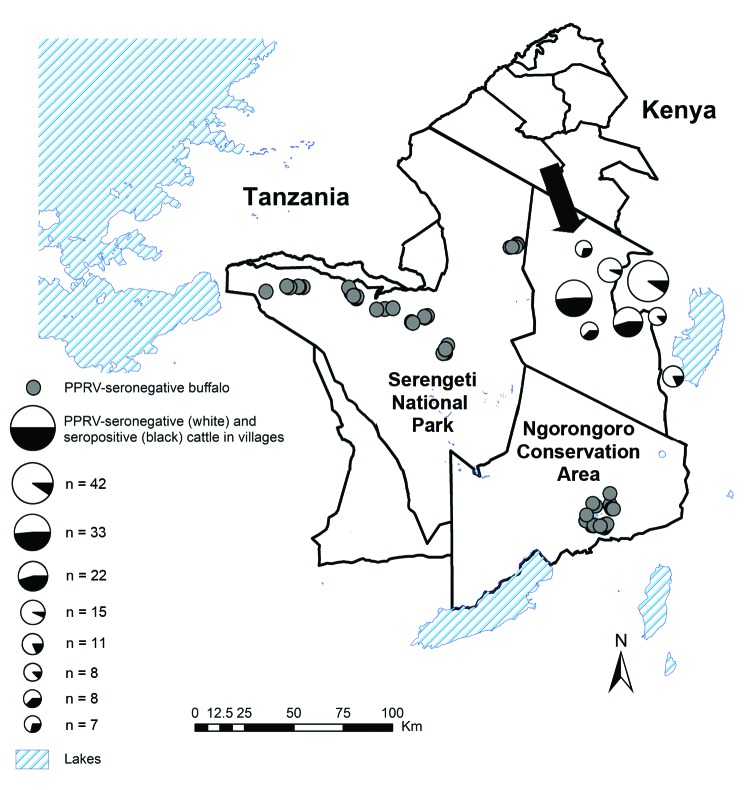
Areas in northwestern Tanzania where seroprevalence of antibodies to peste des petits ruminants virus (PPRV) was studied in cattle and buffalo. Cattle were sampled in 2011; all had been alive during a 2008 PPRV outbreak among small ruminants. Arrow shows location of 1 village affected during the 2008 outbreak (*7*). Buffalo were sampled during 2010–2012 in Serengeti National Park and Ngorongoro Conservation Area; the locations of PPRV-seronegative buffalo are shown.

**Table 1:** Seroprevalence of peste des petits ruminants virus antibodies in cattle and buffalo sampled in northern Tanzania

**Animal, age, y**	**No. positive (%)**	**No. sampled**
Cattle*		
** <1**	0	41
** 1–2**	7 (9.0)	78
** 3**	8 (17.8	45
** 4**	12 (35.3)	34
** 5**	6 (17.6)	34
** 6**	9 (42.9)	21
**>7**	4 (33.3)	12
** Unknown**	0	1
** Total**	46 (17.3)	266
**Buffalo†**		
** 1**	0	5
** 2**	0	17
** 3**	0	23
** 4**	0	10
** 5**	0	26
** 6**	0	11
**>7**	0	58
** Total**	0	150

*Cattle in villages were sampled during 2011. **†*Syncerus caffer.* Buffalo in Serengeti National Park and Ngorongoro Conservation Area were sampled during 2011–2012. Ages were available only for a subset of buffalo.**

Serum samples from buffalo (*Syncerus caffer*) and gazelle (*Eudorcas thomsonii* and *Nanger granti*) came from an archived serum bank and were made available through the Tanzania Wildlife Research Institute–Messerli Foundation Wildlife Veterinary Programme in Seronera, Tanzania. The samples had been collected during wildlife immobilization operations conducted for rinderpest surveillance, research activities, and conservation management (Table 2). Wildlife samples originated from several ecosystems, including the Serengeti ecosystem (Figure). Age information based on incisor tooth eruption was available for a subset of buffalo sampled in the Serengeti National Park and Ngorongoro Conservation Area during 2011–2012 (Table 1).

**Table 2:** Number of wildlife samples from northern Tanzania tested for peste des petits ruminants virus antibodies in northern Tanzania, 2008–2012

**Ecosystem, species**	**No. sampled per year**					**Total no. sampled**
	Before 2008	2008	2010	2011	2012	
Arusha, buffalo*	0	0	0	0	24	24
Katavi, buffalo*	0	0	23	0	0	23
**Ngorongoro Conservation Area**	0	0	0	48	95	143
Buffalo*	0	0	0	0	0	0
** Thomson’s gazelle†**	8	0	0	0	19	27
** Grant’s gazelle‡**	6	0	0	0	0	6
**Serengeti,**	2	3	14	22	10	51
Buffalo*	0	0	0	0	0	0
** Thomson’s gazelle†**	7	0	2	0	23	32
Tarangire, buffalo*	0	0	0	25	0	25
**Total**	23	3	39	95	171	331

*Syncerus caffer. †*Eudorcas thomsonii.* **‡*Nanger granti.***

PPRV antibodies were detected by using the anti-hemagglutinin PPRV C-ELISA (Biological Diagnostic Supplies Limited [BDSL], Dreghorn, UK; www.bdsl2000.com/diagnostic-kits/ppr.aspx). Samples with positive results (i.e., inhibition value >50%) were confirmed as positive by using the anti-nucleoprotein PPRV C-ELISA (IDvet, Grabels, France; www.id-vet.com/produit/id-screen-ppr-competition/). The assays were performed and analyzed according to the manufacturers’ instructions.

The screening for PPRV antibodies in cattle showed that 26.7% of the samples from cattle that were alive during the 2008 PPR outbreak were seropositive, and 5.9% of those from cattle born after the outbreak were seropositive. Seroprevalence in village cattle ranged from 7% to 48% (Figure). No detailed clinical information was available for the period of the outbreak.

Except for 1 borderline positive buffalo sample (inhibition value 56.6%), no seropositive samples were detected among samples from 266 buffalo, 59 Thomson’s gazelles, and 6 Grant’s gazelles. The borderline seropositive buffalo was from the Arusha ecosystem and would have been alive during the 2008 PPR outbreak. PPR-seronegative buffalo included older animals (i.e., >4 years) from Serengeti National Park (n = 20) and Ngorongoro Conservation Area (n = 85) that were alive at the time of the 2008 outbreak and younger animals from Serengeti National Park (n = 10) and Ngorongoro Conservation Area (n = 35).

## Conclusions

Our findings show higher rates of PPR seropositivity in cattle than found in previous studies and confirm that cattle are susceptible to PPR (*1*,*2*). These data support the view that in pastoral communities of northern Tanzania, where small ruminants and cattle co-exist, cross-species transmission of PPRV from small ruminants to cattle is likely to occur frequently.

Two broad conclusions can be drawn from these results. First, cattle are likely to be helpful indicators of PPRV circulation in mixed livestock communities and are therefore a useful population for surveillance. The study indicates that surveillance in cattle may also prove helpful in areas where PPR mass vaccination campaigns in sheep and goats have been implemented and would add value to existing syndromic surveillance networks. This conclusion is supported by the detection of seropositive young cattle (1–2 years of age) in more recent years at a time when no clinical cases were reported in small ruminants in the area. Although transmission of the live attenuated PPR vaccine strain in the field cannot be completely ruled out, there is currently no evidence for vaccine strain transmission either in the field or through experimental infection studies (*14*). Second, the high potential for cross-species transmission of PPRV from small ruminants to cattle in areas where these species live in close proximity suggests that monitoring such livestock communities would be useful for detecting any changes in the apparent pathogenicity of PPRV, including the possible emergence of PPR as a disease in cattle populations.

This preliminary study provided no evidence for PPR infection of wild ruminants within northern Tanzanian ecosystems. However, wildlife are known to be susceptible to PPR (*3*,*12*), and seropositive African buffalo and antelopes have been found in other locations (*15*). This study had limitations that prevent us from drawing definitive conclusions about infection patterns in African wildlife: sampling was largely carried out within wildlife-protected areas where there is limited opportunity for contact with sheep and goats, and only a small number of potential wildlife hosts were sampled. Seroprevalence data from other African buffalo populations (R. Kock, pers. comm.) suggest that larger sample sizes may be required from each host population to detect seropositivity and for our conclusions to be more representative. Thus, we recommend further studies to monitor wildlife infection in populations living in closer proximity to livestock.
